# Prognostic utility of early plasma matrix metalloproteinases -2 and -9 concentrations after severe traumatic brain injury

**DOI:** 10.5935/0103-507X.20200071

**Published:** 2020

**Authors:** Rosane de Lima, Daniel Simon, Willy Deivson Leandro da Silva, Débora Dreher Nabinger, Andrea Regner

**Affiliations:** 1 Postgraduate Program in Cellular and Molecular Biology Applied to Health, Universidade Luterana do Brasil - Canoas (RS), Brazil.; 2 Trauma Biomarkers Laboratory, Universidade Luterana do Brasil - Canoas (RS), Brazil.; 3 Medicine Course, Universidade Luterana do Brasil - Canoas (RS), Brazil.

**Keywords:** Traumatic brain injury, Biomarkers, Matrix metalloproteinase-2, Matrix metalloproteinase-9, Fatal outcome, Mortality, Traumatismo craniencefálico, Biomarcadores, Desfecho fatal, Mortalidade, Metaloproteinase de matriz-2, Metaloproteinase de matriz-9

## Abstract

**Objective:**

To determine whether the matrix metalloproteinases-2 and -9 plasma levels were associated with intensive care unit mortality in patients who suffered severe traumatic brain injury, despite the presence of extracerebral injuries.

**Methods:**

This prospective cohort enrolled 39 male patients who suffered severe traumatic brain injury (Glasgow coma scale: 3 - 8 at hospital admission). The plasma matrix metalloproteinase -2 and matix metalloproteinase -9 levels were determined by ELISA at the time of intensive care unit admission.

**Results:**

Severe traumatic brain injury was associated with a 46% intensive care unit mortality rate. Higher plasma matrix metalloproteinase -9 concentrations were associated with mortality: 147.94 ± 18.00ng/mL for survivors and 224.23 ± 23.86ng/mL for nonsurvivors (mean ± standard error of the mean, p = 0.022). In contrast, there was no significant association between matrix metalloproteinase -2 levels and intensive care unit mortality: 315.68 ± 22.90ng/mL for survivors and 336.55 ± 24.29ng/mL for nonsurvivors (p = 0.499). Additionally, there were no significant associations between matrix metalloproteinase -2 (p = 0.711) and matrix metalloproteinase -9 (p = 0.092) levels and the presence of associated lesions.

**Conclusion:**

Increased plasma matrix metalloproteinase -9 levels were associated with intensive care unit mortality following severe traumatic brain injury, regardless of the presence of extracerebral injuries. Conversely, in this same context, plasma matrix metalloproteinase -2 levels were not associated with short-term fatal outcome prediction.

## Abstract (pt)

**Objetivo:**

Determinar se os níveis plasmáticos das metaloproteinases de matriz -2 e -9 tem associação com a mortalidade na unidade de terapia intensiva em pacientes com trauma craniencefálico grave, independentemente de lesões não cerebrais associadas.

**Métodos:**

Esta coorte prospectiva incluiu 39 pacientes do sexo masculino com trauma craniencefálico grave (escore na escala de coma Glasgow na admissão hospitalar: 3 - 8). Os níveis plasmáticos das metaloproteinases -2 e -9 foram determinados por ELISA no momento da admissão na unidade de terapia intensiva.

**Resultados:**

O trauma craniencefálico grave apresentou mortalidade de 46% na unidade de terapia intensiva. Concentrações mais elevadas de metaloproteinase -9 apresentaram associação com a mortalidade: 147,94 ± 18,00ng/mL para pacientes que sobreviveram e 224,23 ± 23,86ng/mL para os que não sobreviveram (média ± erro padrão, respectivamente; p = 0,022). Todavia, não houve associação significativa entre os níveis de metaloproteinase -2 e a mortalidade na unidade de terapia intensiva: 315,68 ± 22,90ng/mL para o grupo de sobreviventes e 336,55 ± 24,29ng/mL entre os pacientes que não sobreviveram (p = 0,499). Além disso, não se observaram associações significativas entre os níveis de metaloproteinase -2 (p = 0,711) ou metaloproteinase -9 (p = 0,092) e a presença de lesões não cerebrais associadas.

**Conclusão:**

Em vítimas de traumatismo craniencefálico grave, níveis elevados de metaloproteinase -9 tiveram valor preditivo para o desfecho fatal na unidade de terapia intensiva independentemente da presença de lesões não cerebrais associadas. Por outro lado, no mesmo cenário, os níveis plasmáticos de metaloproteinase -2 não apresentaram associação com a mortalidade na unidade de terapia intensiva

## INTRODUCTION

Traumatic brain injury (TBI) is the major cause of death and disabilities in young people worldwide.^(1)^ Severe TBI has been associated with a mortality rate of 30 to 50% (with approximately 90% of deaths occurring within 48 hours of insult) and often causes permanent sequelae.^(2)^ Though the prompt identification of salvageable brain tissue is crucial for the management of patients sustaining severe TBI, the early assessment of injury severity in these patients represents a challenge in intensive care unit (ICU) settings.^(1,3,4)^ Neurotrauma causes damage to the blood-brain barrier (BBB) and, consequently, biomolecules are released into the circulation; this has incited the search for predictive biomarkers that employ minimally invasive sampling techniques and may translate to clinical practice (e.g., S100B, neuron-specific enolase, glial fibrillary acidic protein, and plasma DNA).^(3,5,6)^ Primary injury after severe TBI is caused by mechanical forces that engender deformation of brain tissue. This primary injury triggers a secondary wave of events (i.e., excitoxicity, oxidative stress, metabolic crisis, changes in BBB permeability, inflammatory response, ischemia, and edema), occurring within seconds to minutes after the brain lesion and lasting for days, months, or years.^(1,4,7)^ The ongoing brain damage, which is characteristic of secondary injury, evolves to increased intracranial pressure that can culminate in brain death, particularly in the first 72 hours post-trauma.^(7)^

Matrix metalloproteinases (MMPs) have been implicated in neural injury progression (i.e., BBB breakdown, contusion expansion, and vasogenic edema) following TBI.^(8,9)^ Matrix metalloproteinases form a large family of zinc-dependent endopeptidases related to the dynamic remodulation of the extracellular matrix (ECM). The remodeling of the ECM after neurotrauma has been shown to affect neuronal guidance, synaptic plasticity and regenerative responses.^(8,9)^ Matrix metalloproteinases are finely tuned enzymes that are strategically regulated at the level of transcription, maturation from precursor pro-MMPs, interaction with various ECM components, and inhibition by endogenous inhibitors.^(9)^ Neurons, astrocytes, oligodendrocytes, microglia and endothelial cells have all been shown to express specific MMPs differently after injury, according to brain region, cellular source, and the type of injury.^(9,10)^ Matrix metalloproteinases are also produced by circulating leukocytes that invade the brain during inflammation.^(9)^

Under normal quiescent conditions, MMP expression is very limited, and MMPs are detected either in small amounts or not at all in tissues or the circulation. However, the inflammatory pathway signaling molecules involved in brain injury affect the gene transcription of MMPs.^(7-9)^ Matrix metalloproteinases are upregulated in TBI and can degrade crucial components of the cerebrovascular matrix, leading to disruption of the BBB and exacerbation of edema post-TBI.^(9)^ Several studies in animal models have suggested that MMP-2 and -9, which are inducible MMPs found in the ECM, cerebrospinal fluid, and blood,^(9)^ play important roles in neuroinflammation and secondary injury progression after TBI.^(8,11-15)^ In acute brain injury models, MMP-9 has been shown to have a dual role - a pathological role in BBB disruption, neuronal cell death and hemorrhage early after injury, and a healing role in mediating brain regeneration and neurovascular remodeling during the later repair phase.^(16,17)^

In a cortical contusion model in rats, it was shown that MMP-9 contributes to BBB disruption and brain edema, both of which were attenuated by treatment with the broad-spectrum MMP inhibitor GM6001.^(18)^ Furthermore, MMP-9 knockout mice also exhibited a significant decrease in motor deficits after trauma.^(19)^ In human TBI, several studies investigating a small number of patients have reported higher cerebrospinal fluid (CSF) or circulating MMP-2 and/or MMP-9 levels in patients with TBI.^(8,11,20)^ Recently, studies with larger sample sizes presented controversies regarding the association of plasma MMP-9 levels and mortality in patients with severe TBI. Lorente et al.^(13)^ showed no association of MMP-9 plasma levels and mortality in a cohort of 100 patients. On the other hand, in accordance with the study conducted by Copin et al.,^(12)^ we recently showed an association between plasma MMP-9 levels and ICU mortality.^(21)^ Therefore, there are still controversies regarding the prognostic utility of MMP-2 and MMP-9 levels shortly after severe TBI.

Thus, the aim of this study was to test whether plasma MMP-2 and MMP-9 levels after severe TBI were associated with the primary short-term outcome (ICU mortality) in a series of male patients (isolated or with associated multitrauma). We demonstrated that while MMP-2 did not correlate with ICU mortality, MMP-9 plasma levels may represent a promising predictive biomarker for early prognosis prediction after severe TBI.

## METHODS

Ethical approval of the study protocol (CEP-ULBRA 2008-239H) was granted by the Research Ethics Board of *Universidade Luterana do Brasil*. This study prospectively evaluated a cohort of 39 male patients admitted to the ICUs of three regional trauma centers (*Hospital Cristo Redentor, Hospital de Pronto Socorro de Porto Alegre* and *Hospital de Pronto Socorro Nelson Marchezan de Canoas*) due to severe TBI (Glasgow Coma Scale - GCS 3 - 8 at hospital admission). The patients enrolled had no previous history of neurological or psychiatric disease. On admission to the trauma emergency room, patients were initially evaluated, resuscitated (with crystalloids) and then underwent emergency surgery when necessary. Only patients transferred to the trauma ICU within 12 hours of the head injury were included in the study. The clinical outcome variables for severe TBI included survival (ICU discharge), length of stay in the ICU and neurological assessment using the GCS at admission and Glasgow Outcome Scale (GOS) at ICU discharge. At admission to the trauma ICU, circulatory function and GCS scores were monitored. All patients were sedated and mechanically ventilated, and corticosteroids were not administered. Previous studies established sex differences in the pathophysiology and outcome after acute neurological injury.^(22)^ Less susceptibility to postischemic and posttraumatic brain injury in females has been observed.^(22)^ Thus, to avoid interference from possible sex-dependent differences in outcome following brain trauma, only males were enrolled in the study.

### Blood sampling and plasma MMP-2 and -9 determinations

Peripheral venous blood was collected into heparin-containing tubes at ICU admission. Blood samples were centrifuged at 1000g for 10 minutes, and then the plasma was removed (with great care taken not to disturb the pellet) and frozen in aliquots at -20°C until batch evaluation. Plasma MMP-2 and MMP-9 concentrations were determined by ELISA (Human MMP-2 and MMP-9 kits, Invitrogen, California, USA).

### Statistical analysis

Continuous variables were compared between groups using Student’s t-test or the nonparametric Mann-Whitney U test. Categorical variables were tested using the chi-square test. Correlations were analyzed using Spearman´s nonparametric correlation method or linear regression method. The extent to which the plasma MMP-9 concentrations differed between individuals surviving or dying in the ICU after severe TBI was assessed using receiver operator characteristics (ROC) plots. The ROC plot was obtained by calculating the sensitivity and specificity for every distinct observed data value and plotting sensitivity against 1-(specificity). The ROC curve was used to evaluate the optimal cut-off values measured at study entry for predicting an unfavorable outcome. A cut-off point on the curves was chosen to attain the best compromise between sensitivity and specificity for death in the ICU. Logistic regression analysis was performed to eliminate the effect of confounding factors, and the dependent variable was the primary outcome (dead/alive). The independent variables tested were age, prehospital care, associated injury, craniotomy, GCS score at hospital admission, infection during ICU stay and plasma MMP-9 levels. All p values presented are two-tailed, and p < 0.05 was considered statistically significant.

## RESULTS

### Characteristics of the traumatic brain injury population

This study included 39 male patients who suffered severe TBI. Table 1 shows the characteristics of the TBI patients stratified according to primary outcome measure (ICU mortality). Severe TBI was associated with a 46% mortality rate, and the median time between traumatic events and death was 4 days; most occurred within 72 hours after ICU admission. The median age of the patients was 30 years, and there was no significant difference in age between the patients who survived (median 27 years) and the patients who did not (median 33 years). Most patients (84%) received prehospital care. Surviving patients were admitted to the hospital with GCS scores of 6.35 ± 1.69 (mean ± S.D.), while those who had fatal outcomes had significantly lower GCS scores (4.66 ± 1.97; p = 0.013). A total of 15 (38.5%) patients underwent craniotomy. The length of stay in the ICU ranged from 1 to 40 days, with a significant difference when comparing survivors (median 17, range 4 - 40) and nonsurvivors (median 4.0, range 1 - 22 days; p < 0.001). The mean GCS and GOS scores at ICU discharge were 10.47 ± 4.18 and 2.95 ± 1.01, respectively. The main mechanism of injury was related to motor vehicle accidents (46%), followed by interpersonal violence (26%). Twenty-three patients (59%) presented associated injuries; however, there was no significant difference between the presence of multisystem trauma and ICU mortality (p = 0.088) (Table 1).

**Table 1:** Traumatic brain injury study population stratified by the primary outcome measure (intensive care unit mortality)

Variables	All patients (n = 39)	ICU discharge (n = 21)	ICU mortality (n = 18)	p value
Age (years)	30 (18 - 64)	27 (18 - 62)	33 (21 - 64)	0.146
Prehospital care	34 (87.2)	20 (95.2)	14 (77.8)	0.050
GCS at hospital admission	5.55 ± 1.99	6.35 ± 1.69	4.66 ± 1.97	0.013*
GCS at ICU admission	5.46 ± 2.93	6.47 ± 3.35	4.33 ± 1.88	0.050
Systolic blood pressure (mmHg)	131 (60 - 190)	134 (74 - 180)	130 (30 - 190)	0.796
Diastolic blood pressure (mmHg)	77 (30 - 118)	77 (34 - 108)	80 (30 - 118)	0.410
Time to blood sampling (hours after hospital admission)	6.4 ± 5.5	7.2 ± 6.2	5.5 ± 4.5	0.376
Mechanism of injury				0.508
Motor vehicle accident	18 (46.2)	12 (57.1)	6 (33.3)	
Auto versus pedestrian	7 (17.9)	3 (14.3)	4 (22.2)	
Fall	4 (10.3)	2 (9.5)	2 (11.1)	
Assault	10 (25.6)	4 (19.1)	6 (33.3)	
Craniotomy	15 (38.5)	9 (42.9)	6 (33.3)	0.463
Associated injuries	23 (59.0)	15 (71.4)	9 (50.0)	0.088
Infection during ICU stay	26 (66.7)	17 (80.9)	9 (50.0)	0.041*
Outcome time (days)	9.5 (1 - 40)	17.0 (4 - 40)	4.0 (1 - 22)	< 0.001*
GCS at discharge from ICU		10.47 ± 4.18		
GOS at discharge from ICU		2.95 ± 1.01		
Plasma MMP-2 (ng/mL)	325.31 ± 16.53	315.68 ± 22.90	336.55 ± 24.29	0.499
Plasma MMP-9 (ng/mL)	183.15 ± 15.73	147.94 ± 18.00	224.23 ± 23.86	0.002*

ICU - intensive care unit; GCS - Glasgow Coma Scale; GOS - Glasgow Outcome Scale; MMP - matrix metalloproteinase.

* Statistically significant (Mann-Whitney or Chi-square tests). Results median (range), n (%), mean ± standard deviation or mean ± standard error of the mean.

Characteristics of the TBI population stratified by the type of severe TBI (isolated TBI or TBI associated with multitrauma) are depicted in table 2. There were no significant differences in age, GCS scores at either emergency room or ICU admission, blood pressure at hospital admission, time between trauma and outcome, or GCS scores at ICU discharge between the groups (Table 2). However, we observed a trend (p *=* 0.058) of lower GCS at ICU discharge in patients who suffered multisystem trauma associated with TBI (Table 2).

**Table 2:** Characteristics of the traumatic brain injury study population stratified by the type of traumatic brain injury (isolated/associated with multitrauma)

Variable	Isolated (n = 16)	Multitrauma (n = 23)	p value
Age (years)	30.5 (18 - 64)	30.0 (18 - 62)	0.819
Prehospital care	13 (81.2)	21 (91.3)	0.336
GCS at hospital admission	5.12 ± 2.12	5.86 ± 1.88	0.297
GCS at ICU admission	5.30 ± 2.21	5.57 ± 3.38	0.839
Systolic blood pressure (mmHg)	140 (60 - 184)	127 (74 - 190)	0.300
Diastolic blood pressure (mmHg)	86 (34 - 118)	75 (30 - 113)	0.222
Mechanism of injury			0.172
Motor vehicle accident	4 (25.0)	14 (60.9)	
Auto versus pedestrian	4 (25.0)	3 (13.0)	
Fall	2 (12.5)	2 (8.7)	
Assault	6 (37.5)	4 (17.39)	
Time to blood sampling (hours after hospital admission)	5.60 ± 4.70	6.97 ± 6.05	0.222
Craniotomy	6 (37.5)	19 (82.6)	0.832
Infection during ICU stay	9 (56.3)	17 (73.9)	0.250
Outcome time (in days)	6 (1 - 39)	13 (2 - 40)	0.080
GCS at discharge from ICU	13.33 ± 5.04	9.15 ± 3.10	0.058
GOS at discharge from ICU	1.94 ± 1.44	2.05 ± 1.12	0.794
Plasma MMP-2 (ng/mL)	316.59 ± 25.57	331.38 ± 22.06	0.711
Plasma MMP-9 (ng/mL)	197.89 ± 24.53	172.90 ± 20.68	0.092

GCS - Glasgow Coma Scale; ICU - intensive care unit; GOS - Glasgow Outcome Scale; MMP - matrix metalloproteinase. Results median (range), n (%), mean ± standard deviation or mean ± standard error of the mean.

### Plasma MMP-2 and MMP-9 levels

Plasma MMP-2 and MMP-9 levels were determined early after TBI at ICU admission (mean 6.4 ± 5.5 hours after hospital admission). The mean plasma MMP-2 concentration in patients with severe TBI was 325.31 ± 16.53ng/mL (mean ± S.E. M) (Table 1). There was no significant difference between MMP-2 levels in the survivor (315.68 ± 22.90ng/mL) and nonsurvivor (336.55 ± 24.29ng/mL) groups (p = 0.499) (Table 1). Besides, there was no correlation between MMP-2 levels and GOS scores at ICU discharge (Spearman´s rho = -0.172, p = 0.309). Additionally, regarding the type of lesion, there was no significant difference (p = 0.711) in plasma levels of MMP-2: 316.59 ± 25.57ng/mL in the isolated TBI and 331.38 ± 22.06ng/mL in the TBI associated with multitrauma groups (mean ± standard error of the mean - SEM) (Table 2).

The mean plasma MMP-9 concentration in patients with severe TBI was 183.15 ± 15.73ng/mL (mean ± SEM) (Table 1). Notably, there was a significant difference between MMP-9 levels in the survivor (147.94 ± 18.00ng/mL) and nonsurvivor (224.23 ± 23.86ng/mL) groups (p = 0.002, Mann-Whitney U test) (Table 1). Indeed, there was a correlation between higher levels of MMP-9 and ICU mortality (Spearman´s rho = 0.498, p = 0.001). Additionally, there was also a correlation between MMP-9 levels and the length of stay in the ICU (linear regression, p = 0.020) and GOS scores at ICU discharge (Spearman´s rho = -0.491, p = 0.002). In contrast, there was no significant difference between MMP-9 levels and the type of lesion: 197.89 ± 24.53ng/mL for patients with isolated TBI and 172.90 ± 20.68ng/mL for patients with associated extracerebral injuries (mean ± standard error of the mean - SEM, p = 0.092) (Table 2). Furthermore, there were no correlations between MMP-9 levels and craniotomy (Spearman´s rho = -0.160, p = 0.339) or infection during the ICU stay (Spearman´s rho = -0.101, p = 0.539). ROC curves were plotted, and a cut-off point that would ensure the detection of the highest proportion of individuals with fatal outcomes with the least compromise in specificity was chosen. Therefore, a cut-off point of 150.1ng/mL of MMP-9 within 12 hours after hospital admission was chosen. The diagnostic characteristic of this cut-off point was a specificity of plasma MMP-9 concentration for predicting mortality of 86% and a sensitivity of 72%. The area under the curve for MMP-9 plasma concentration was 0.788 (95% confidence interval - 95%CI: 0.640 - 0.936; p = 0.002).

Logistic regression analysis was performed to assess the independent influence of MMP-9 plasma levels on the TBI primary outcome (ICU mortality). After adjusting for confounding variables, we found that lower GCS at hospital admission (p = 0.049), infection during the ICU stay (p = 0.031) and plasma MMP-9 levels (p = 0.011) were variables independently associated with poor outcome (death).

## DISCUSSION

Considering that the correct and timely diagnosis of deterioration in severe TBI remains a major challenge in clinical practice, we studied plasma MMP-2 and MMP-9 levels in 39 male patients at ICU admission; levels were assessed in the early phase after severe TBI (mean 6.4 hours after hospital admission). The study showed an association between higher plasma MMP-9 levels and ICU mortality, despite the presence of associated multitrauma. In contrast, there was no significant association between MMP-2 levels and the short-term fatal outcome. In accordance with the literature, patients enrolled in our study were mostly young men involved in traffic accidents and interpersonal violence.^(1)^ The intensive care unit mortality rate was 46%, and lower GCS scores at hospital admission, infection during the ICU stay and plasma MMP-9 levels were independently associated with poor outcome (death).

In the context of TBI, MMPs are involved in rapidly progressing brain injury, typically leading to BBB disruption, cell death and brain edema.^(15)^ Matrix metalloproteinases are also thought to play important roles in cell proliferation, migration (adhesion/dispersion), differentiation, angiogenesis, synaptogenesis, apoptosis, and host defense.^(9)^ In addition to these functions, MMPs act on proinflammatory cytokines to regulate various aspects of neuroinflammation following TBI.^(23)^ Brain injury activates microglia that, in turn, may release MMP-2, -9 and IL-6.^(24)^ In fact, Suehiro et al.^(25)^ reported that hypothermia can be neuroprotective by reducing postinjury increases in MMP-9 and IL-6. Furthermore, Harkness et al.^(26)^ showed that *in vitro* activation of brain microvascular endothelium with proinflammatory cytokines, such as tumor necrosis factor-alpha (TNF-alpha), results in a selective upregulation of the expression of MMP-9. Notably, our group has reported increased levels of circulating Fas, TNF-alpha and IL-6 early after severe TBI.^(27,28)^ Additionally, Kim et al.^(29)^ showed that Hsp70 knockout mice subjected to TBI present increased lesion size, worsened brain hemorrhage and increased expression and activation of MMPs. Notably, in a previous study, we demonstrated a correlation between serum Hsp70 concentrations and outcome after severe TBI.^(30)^

In animal studies, evidence shows that MMP-2 and MMP-9 contribute to secondary injury progression following neurotrauma.^(14,15,31,32)^ Wang et al.^(19)^ demonstrated that in knockout mice deficient in MMP-9, gene expression indicated reduced morphological damage in a model of TBI. Furthermore, downregulation of MMP-9 attenuated brain edema after TBI,^(33)^ whereas SB-3CT, a potent and selective inhibitor of MMP-2 and MMP-9, reduced secondary injury progression and improved long-term neurobehavioral outcomes post-TBI.^(34)^ Recently, Pijet et al.^(35)^ showed the contribution of MMP-9 to long-term structural and physiological alterations in brain circuitry post-TBI. Nonetheless, the time course and peak of MMP-2 and MMP-9 expression and activation following acute brain injury are still uncertain; elevation in the brain may begin as early as 10 minutes after trauma and persist for over 7 days.^(15,19)^

Concerning human TBI, Vilalta et al.^(8)^ observed elevated levels of pro-MMP-2 and pro-MMP-9 in the plasma and CSF of 20 patients 12 hours after TBI. Grossetete et al.^(11)^ reported high levels of MMP-9 in the CSF of 7 patients early after severe TBI. Additionally, Liu et al.^(36)^ reported that early determination of MMP-9 concentrations in the CSF of 6 TBI patients correlated with prognosis. Suehiro et al.^(25)^ found elevated levels of circulating MMP-9 at hospital admission in patients with TBI, while Vajtr et al.^(20)^ investigated 18 patients and reported that higher levels of plasma MMP-9 were found during the first 3 days in patients who underwent decompressive neurosurgery following TBI. More recently, Lorente et al.^(13)^ investigated serum levels of MMP-9 in a large cohort of 100 patients with severe TBI at hospital admission and did not show an association of MMP-9 with 30-day mortality. In contrast, Copin et al.^(12)^ demonstrated, in a cohort of 49 patients, that MMP-9 concentrations predicted death in the first 48 hours after severe TBI. Notably, in 2017, in a cohort of 80 patients with severe TBI, we also showed that increased plasma MMP-9 levels predicted short-term fatal outcomes, regardless of the presence of extracerebral injuries.^(21)^

Accordingly, in the present study, we found that early higher plasma MMP-9 levels were associated with ICU mortality. Conversely, MMP-2 plasma levels did not predict short-term fatal outcomes. This observed divergence in the prognostic utility of MMP-2 and MMP-9 is in conformity with previous studies. Shi et al.^(37)^ investigated early changes in the concentration of MMP-2, -9 and tissue inhibitor of metalloproteinase (TIMP-1 ) in a rat model of brain injury combined with trauma-induced heterotopic ossification and showed that MMP-9, but not MMP-2, contributed to the remodeling and calcification of the ECM, resulting in the induction of osteoblast precursor cells in heterotopic ossification. In a focal injury model, Guilfoyle et al.^(38)^ reported that, though MMP-9 concentrations were elevated in pericontusional brain tissue compared to normal brain tissue, no signiﬁcant difference was found for MMP-2. Moreover, Underly et al.^(39)^ showed that coinjection of pericyte somata and a MMP-9 inhibitor, but not a MMP-2 inhibitor, was able to reduce BBB damage during cerebral ischemia. Distinct patterns of expression and activation of MMP-2 and MMP-9 may at least partially explain their different roles after TBI.^(8,40)^

Overall, studies have indicated that both local and systemic trauma-induced upregulation of MMP-2 and MMP-9 during the acute phase postinjury are implicated in the pathophysiology of TBI.^(8,14-19,25,33,40)^ For this reason, MMPs represent promising therapeutic targets. In effect, robust evidence has shown that pharmacological inhibitors of MMPs can reduce TBI-mediated edema formation, BBB impairment, inflammatory responses, and cerebral ischemia.^(14-19,25,33)^ Alternatively, it is important to consider the apparent positive effects of MMP-2 and MMP-9 in repair and regeneration after nervous system injury.^(9,36)^ As a matter of fact, Danilina et al.^(41)^ studied the neuroprotective potential of multipotent mesenchymal stromal cells exposed to inflammatory preconditioning in TBI. Culture conditions simulating inflammation increased the production of MMP-2 and MMP-9 but did not reduce their therapeutic effectiveness. Moreover, in some variants of inflammatory preconditioning, mesenchymal cells exhibited more pronounced neuroprotective properties, reducing the volume of brain lesions and promoting recovery of neurological functions after TBI.^(41)^

Although we demonstrated that levels of plasma MMP-9 predicted short-term mortality and GOS scores following severe TBI, possible limitations of the present study were the limited sample size and the fact that we did not analyze the potential predictive value of MMP-2 or MMP-9 considering longer-term outcomes of disability and mortality.

## CONCLUSION

In the present study, we demonstrated that higher plasma MMP-9, but not MMP-2, concentrations predicted short-term intensive care unit mortality and Glasgow Outcome Scale scores at discharge following severe traumatic brain injury regardless of the presence of associated multitrauma. Therefore, the translational potential of MMP-9 after traumatic brain injury is promising in both the diagnostic and treatment domains.
